# CD71+ erythroid cells as a potential early biomarker for hemodynamic significant patent ductus arteriosus in preterm infants

**DOI:** 10.3389/fimmu.2025.1738166

**Published:** 2026-01-13

**Authors:** Ju Ae Shin, Jae Young Lee, Jeongmin Shin, Min Soo Kim, Young-Ah Youn, Joungok Kim, Eun-Jee Oh, Jin-Hee Oh

**Affiliations:** 1 Department of Pediatrics, Seoul St. Mary’s Hospital, College of Medicine, The Catholic University of Korea, Seoul, Republic of Korea; 2 Department of Laboratory Medicine, Seoul St. Mary’s Hospital, College of Medicine, The Catholic University of Korea, Seoul, Republic of Korea; 3 Research and Development Institute for In Vitro Diagnostic Medical Devices of Catholic University of Korea, Seoul, Republic of Korea; 4 Department of Pediatrics, St. Vincent’s Hospital, College of Medicine, The Catholic University of Korea, Seoul, Republic of Korea

**Keywords:** CD71+ erythroid cell, hemodynamically significant patent ductus arteriosus, immunoregulation, patent ductus arteriosus, preterm infants

## Abstract

**Introduction:**

Hemodynamically significant patent ductus arteriosus (hsPDA) in preterm infants is associated with increased mortality and morbidity. Early and accurate identification of infants at risk for PDA is critical for improving outcome. Perinatal inflammation is a known risk factor of PDA. CD71+ erythroid cells (CECs) are immature red blood cells with immunomodulatory roles and show variable abundance in preterm infants. We aimed to examine the proportion of CECs in preterm infants and investigate association with PDA.

**Methods:**

In this prospective study, we evaluated the association between CEC levels and the development of hsPDA in a cohort of 108 preterm infants born before 37 weeks of gestation. CEC levels were quantified by flow cytometry within 12 hours of birth. The presence of hsPDA was assessed by echocardiography between days 3 and 7 after birth.

**Results:**

CEC levels showed a significant inverse relationship with gestational age (CEC(%) = -0.4419 × Gestational Age + 18.7029, p<0.001). CEC levels were also inversely correlated with hemoglobin, red blood cells, white blood cells, and platelets (all *p* < 0.05). Infants who developed hsPDA had significantly higher CEC levels (7.5 ± 2.8% vs. 4.4 ± 1.7%, *p* < 0.0001), together with lower values of these hematologic parameters than infants without hsPDA. Multivariable logistic regression identified elevated CECs levels (odds ratio: 2.048, 95% CI: 1.326–3.162, *p* = 0.0012) and lower white blood cell count (odds ratio: 0.782, 95% CI: 0.620–0.986, *p* = 0.038) as independent predictors of hsPDA. Additionally, CECs levels were inversely correlated with hemoglobin, red blood cells, white blood cells, and platelets (p<0.05).

**Discussion:**

These results suggest that early measurement of CEC levels can identify preterm infants at high risk for hsPDA prior to echocardiographic diagnosis. As a potential predictive biomarker, CECs may improve risk stratification and guide timely intervention, ultimately enhancing clinical outcomes. Further research is needed to elucidate the mechanisms linking CECs to hsPDA.

## 1 Introduction

Persistent patent ductus arteriosus(PDA) in preterm infants is associated with various neonatal morbidities, including bronchopulmonary dysplasia, intraventricular hemorrhage, pulmonary hemorrhage, necrotizing enterocolitis (1). Unlike full-term infants, the incidence of PDA exceeds 50% in preterm infants born at less than 28 weeks’ gestation (2). Closure of the ductus arteriosus is influenced by a variety of physiologic, molecular, structural factor (3). Among these, prostaglandin E2(PGE2) plays a key role as a potent vasodilator. During fetal development, PGE2 levels remain high, but, they rapidly decline after birth due to decreased placental production and increased catabolism in the lungs (4). Pharmacologic closure of PDA primarily targets prostaglandin inhibition. Inflammation is increasingly recognized as a contributing factor in PDA. Chorioamnionitis, which induces a fetal inflammatory response, is associated with an increased risk of PDA (5). Conversely, antenatal steroids with anti-inflammatory effects reduce the risk of PDA (6). Elevated inflammatory biomarkers in the umbilical cord, such as IL-6, IL-8, IL-10, and IL-12, have also been associated with PDA development (7).

PDA represents a major challenge in neonatal care. While small PDA does not usually affect the hemodynamic circulation in preterm infants, larger ones can cause pulmonary overcirculation and decreased systemic perfusion, termed hemodynamically significant PDA(hsPDA). Currently, the terms PDA and hsPDA are often used interchangeably in preterm infants. Recent studies suggest that early extubation in preterm infants improves outcomes by reducing hospital stay, mortality, bronchopulmonary dysplasia, and intraventricular hemorrhage (8, 9), whereas extubation failure—frequently associated with hsPDA—has a markedly adverse impact on prognosis (10). In addition, fluid restriction, which is often employed as a conservative strategy for hsPDA, may impair nutrition, growth, and organ perfusion. Therefore, early identification of hsPDA is crucial to guide timely, individualized treatment strategies and ultimately improve neonatal outcomes. Although echocardiography remains the diagnostic gold standard, its interpretation depends on operator expertise and clinical context, such as the presence of an atrial septal defect or patent foramen ovale and intravascular volume status. Predicting hsPDA development based solely on the clinical presentation at birth remains difficult, highlighting the need for reliable early biomarkers and novel predictive approaches.

CD71+ erythroid cells (CECs) are immature red blood cells, including erythroblasts and reticulocytes, characterized by co-expressing transferrin receptor 1 (CD71) and erythroid lineage markers, glycophorin A (CD235a) in humans (11). While CECs are almost exclusively found in the bone marrow of adults, they are abundant in the peripheral blood of neonates but decrease with age (12, 13). Beyond erythropoiesis, CECs possess immunomodulatory functions that influence both innate and adaptive immune responses (11, 12). The immunosuppressive effects contribute to neonates’ increased susceptibility to infections but also facilitate colonization of commensal microorganisms after birth (11, 14). B-type natriuretic peptide (BNP) and NT-proBNP are well-established cardiac biomarkers that reflect myocardial wall stress in response to volume overload and are used in diagnosing hsPDA. However, BNP levels typically peak at 48–72 hours after birth and require serial measurements for reliable interpretation. In contrast, CECs reflect endothelial activation—an upstream pathophysiological response to perinatal hemodynamic stress and vascular injury. Therefore, investigating CECs measured within the first 12 hours of life as an early biomarker may offer additional value for very early risk stratification of infants who will subsequently develop hsPDA.

An early blood-based biomarker that identifies preterm infants at high risk for hsPDA before echocardiographic confirmation could improve clinical decision-making by enabling risk stratification and more timely, individualized treatment. Given the established link between inflammation and hsPDA and the immunomodulatory role of CECs, we hypothesized that circulating CEC levels in the first day of life are associated with the subsequent development of hsPDA in preterm infants and may serve as a predictive biomarker. In addition, hemodynamic stress and tissue hypoxia associated with hsPDA may stimulate compensatory erythropoiesis, potentially altering circulating CEC populations. Therefore, in this study we aimed to examine whether the abundance of CECs is associated with hsPDA in preterm infants. To address this, we measured CEC levels in a single peripheral blood sample collected within 12 hours of birth and examined how these values varied with gestational age, compared CEC levels between infants with and without hsPDA, and evaluated whether early CEC measurement could serve as a predictive biomarker for hsPDA development.

To our knowledge, no previous animal or human studies have explored the association between CD71+ erythroid cells and hsPDA in preterm infants.

## 2 Materials and methods

### 2.1 Study population

We prospectively and consecutively enrolled preterm infants (< 37 weeks’ gestation) admitted to the neonatal intensive care unit(NICU) of our institution between January 2024 and September 2024. This study followed the principles of the Declaration of Helsinki and was approved by the Institutional Review Board of Seoul St. Mary’s Hospital (IRB no. KC23TISI0899). Informed consent was obtained from the parents of all study participants. Exclusion criteria included lack of parental consent, major congenital anomalies, chromosomal or genetic anomaly, and critical intrauterine or perinatal illness, and difficulty in obtaining a blood sample within 12 hours after birth.

### 2.2 Data collection

Gestational age, birth weight, birth-weight below the 10^th^ percentile for the specific gestational age based on the Korean newborn survey and statistics (15), sex, mode of delivery, 1- and 5-min Apgar score, multiple gestation, maternal history and perinatal morbidities were collected through medical record review. Laboratory data including blood gas analysis, complete blood count and C-reactive protein (CRP) at birth, were also obtained. Assessment for hsPDA was performed by experienced pediatric cardiologists between 3 and 7 days after birth. This timing was chosen because hemodynamically significant PDA typically becomes apparent only after pulmonary vascular resistance falls, a process that begins around day 3 of life. Echocardiography was performed using an Affiniti 50 (Philips Ultrasound, Bothell, Seattle, WA) and a pediatric cardiac and neonatal head probe (12-14MHz). If clinical symptoms such as pulmonary hemorrhage, hypotension, decreased urine output, feeding intolerance, abdominal distension, or high ventilation pressure requirements were observed, echocardiography was conducted earlier, between day 3 and 4 after birth. Asymptomatic infants underwent routine echocardiography within the first week for hsPDA screening. hsPDA was defined by meeting the E3 or E4 echocardiographic criteria according to the staging system by McNamara and colleagues (16) (**Supplementary Table 1**).

### 2.3 CD 71+ RBC analysis using flow cytometry

**Figure 1:**
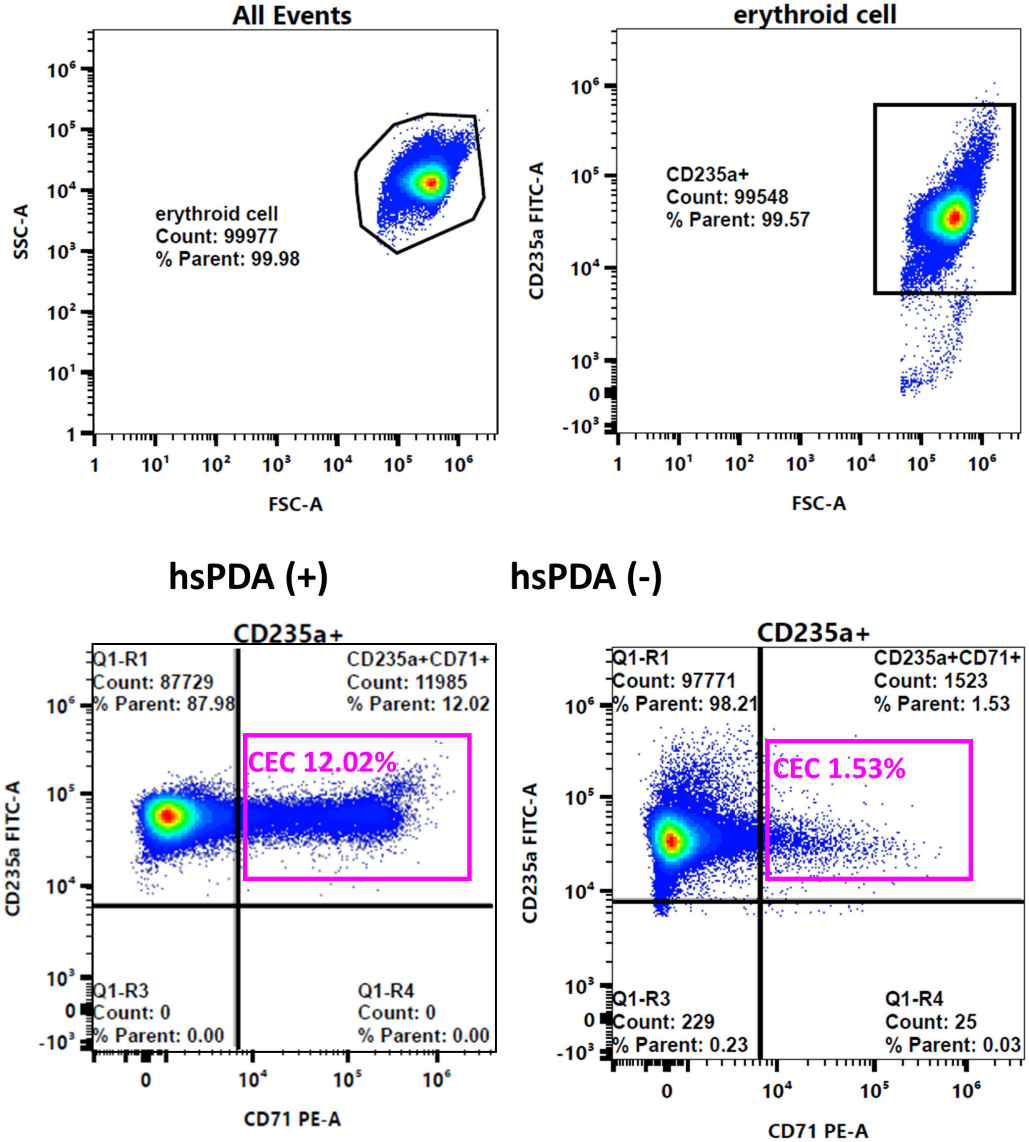
Representative flow cytometry analysis of CD71+ erythroid cells (CECs) in peripheral blood from preterm infants with and without hemodynamically significant patent ductus arteriosus (hsPDA). Upper panels: Gating strategy for the identification of erythroid cells, defined by forward scatter (FSC-A), side scatter (SSC-A), and CD235a expression. Lower panels: Quantification of CECs (CD235a+CD71+) as a percentage of total red blood cells (RBCs) in hsPDA-positive (left, CES 12.02%) and hsPDA-negative (right, CES 1.53%) infants.

Peripheral blood samples were collected in K3 EDTA tubes (Becton/Dickinson, San Jose, CA). For each sample, 200 μL was used and processed within 48 hours, stored at 4 ± 2°C prior to analysis. Blood for CEC measurement was drawn at the same time as routine laboratory tests (Hb, RBC, WBC, and blood gas) performed within the first 12 hours after birth. Samples were diluted with phosphate-buffered saline (PBS), and stained with anti-CD235a-FITC and anti-CD71-PE monoclonal antibodies (Beckman Coulter, Brea, CA, USA). After staining, cells were washed and resuspended in 500 μL of PBS for analysis. Flow cytometry was performed using a Cytek Northen Lights flow cytometer (Cytek Biosciences). Red blood cells (RBCs) were selected based on forward scatter and side scatter, and a total of 100,000 RBCs were analyzed per sample. Total RBCs were gated using CD235, and CECs were quantified as the percentage of CD235+ CD71+ cells among total RBCs. Data were analyzed using SpectroFlo software (Cytek Biosciences, Fremont, CA, USA) (**Figure 1**). To ensure analytical reliability and consistency, all CEC measurements were performed by a single trained operator using unstained RBCs as the reference to establish the positive population, and the turnaround time was approximately 24–48 hours. When measurements were repeated, the difference between paired values was less than 5%.

### 2.4 Statistical analysis

All statistical analyses were conducted using SAS version 9.4 (SAS Institute Inc., Cary, NC, USA), Prism 10 (GraphPad Software, San Diego, CA). Continuous variables are presented as mean ± standard deviation, and categorical variables as number and percentage. Group differences were evaluated using independent t-tests or Mann-Whitney U tests for continuous variables, and Chi-square tests or Fisher’s exact tests for categorical variables. Bonferroni correction was applied for multiple comparisons. Simple linear regression was used to assess the relationship between CECs and gestational age. Correlations between gestational age and other hematologic parameters were also evaluated. Univariable and multivariable logistic regression analyses with backward elimination were used to identify hsPDA predictors. Variables with a *p* < 0.02 in univariable analysis were included in the multivariable model. Receiver operating characteristic (ROC) curve analysis was performed to assess the predictive performance of CEC levels for hsPDA in the overall cohort and in the subgroup with gestational age ≤28 weeks. A p-value < 0.05 was considered statistically significant.

## 3 Results

### 3.1 Baseline characteristics

The baseline characteristics of the 108 preterm infants enrolled in this study are summarized in **Table 1**. Of these, 98 infants had either no PDA (n = 88) or restrictive PDA (n = 10), while 10 infants were diagnosed hsPDA. The hsPDA group had significantly lower gestational age compared to the no/restrictive PDA group (27.2 ± 3.6 weeks vs. 32.2 ± 3.0 weeks, *p* < 0.001). As expected, birth weight and both 1- minute and 5-minute Apgar score were also significantly lower in the hsPDA group (all *p* < 0.05). There were no significant differences in the incidence of premature rupture of membrane (PROM), histologic chorioamnionitis or antenatal corticosteroid use between the groups. Other characteristics were similar between the two groups.

**Table 1:** Baseline characteristics of preterm infants by hsPDA status.

Parameter	Total (n=108)	hsPDA (–) (n=98)	hsPDA(+) (n=10)	P-value
Gestational age (weeks), mean ± SD (range)	31.8 ± 3.4 (22.3 – 36.9)	32.2 ± 3.0 (22.3 – 36.9)	27.2 ± 3.6 (22.3 – 34.7)	<0.0001
Gestational age ≥30.0 weeks, n (%)	75 (69.4)	73 (74.5)	2 (20.0)	0.0011
Gestational age <26.0 weeks, n (%)	5 (4.6)	2 (2.0)	3 (30.0)	0.0001
Small for GA, <10th percentile, n (%)	25 (23.2)	23 (23.5)	2 (20.0)	1.0000
Male, n (%)	63 (58.3)	57 (58.2)	6 (60.0)	1.0000
Cesarean delivery, n (%)	102 (94.4)	94 (95.9)	8 (80.0)	0.0950
Multiple births, n (%)	37 (34.3)	33 (33.7)	4 (40.0)	0.7331
Rupture of membrane, n (%)	22 (20.4)	18 (18.4)	4 (40.0)	0.1171
Pregnancy-induced hypertension, n (%)	24 (22.2)	21 (21.4)	3 (30.0)	0.6893
Maternal diabetes, n (%)	14 (13.0)	11 (11.2)	3 (30.0)	0.1204
Placenta abruption, n (%)	2 (1.9)	2 (2.0)	0 (0.0)	1.0000
Meconium stained, n (%)	3 (2.8)	3 (3.1)	0 (0.0)	1.0000
Histologic chorioamnionitis, n (%)	18 (16.7)	15 (15.3)	3 (30.0)	0.3654
Antenatal corticosteroids, n (%)	99 (91.7)	89 (90.8)	10 (100.0)	1.0000
Delayed cord clamping, n (%)	0 (0.0%)	0 (0.0%)	0 (0.0%)	–
Maternal age (years), mean ± SD	35.0 ± 3.4	35.0 ± 3.3	34.3 ± 4.3	0.5114
Birth weight (g), mean ± SD	1758.1 ± 650.8	1833.0 ± 619.4	1023.6 ± 489.7	0.0001
Apgar score at 1 min, mean ± SD	5.6 ± 1.7	5.8 ± 1.6	3.4 ± 2.0	<0.0001
Apgar score at 5 min, mean ± SD	8.1 ± 1.1	8.2 ± 1.0	6.7 ± 1.2	<0.0001

### 3.2 Relationship between gestational age and CEC levels

**Figure 2:**
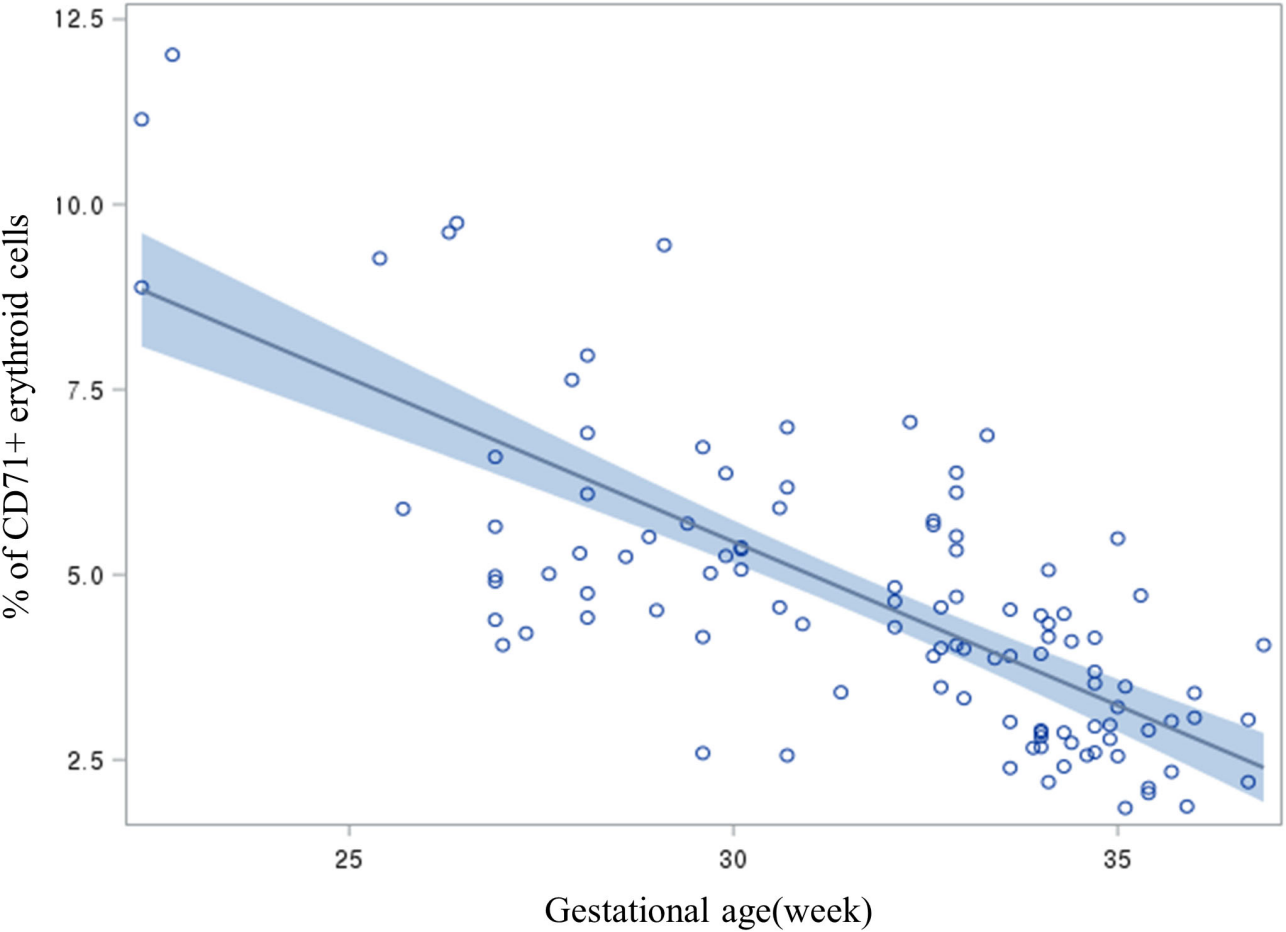
Scatter plot and linear regression illustrating the inverse relationship between gestational age and circulating CD71+ erythroid cell (CEC) percentage in preterm infants. CEC(%) = -0.4419 × Gestational Age + 18.7029, p < 0.001). Each point represents an individual neonate’s data; the blue regression line and shaded confidence interval highlight a significant decrease in CECs with increasing gestational age (p < 0.001; n = 108).

**Figure 2** depicts the relationship between gestational age and CEC percentage in preterm infants using a scatter plot with regression analysis. There was a significant inverse relationship between gestational age and CEC percentage (CEC(%) = -0.4419 × Gestational Age + 18.7029, p<0.001). Specifically, CEC (%) decreased by an average of 0.44 for each 1-week increase in gestational age. For example, preterm infants born at 25 weeks’ gestation had average CEC levels of approximately 7.65%, whereas those born at 35 weeks’ gestation had levels around 3.24%. This finding indicates that CEC levels decrease steadily as gestational age increases.

### 3.3 Comparison of hematologic parameters and CECs by hsPDA status

**Figure 3:**
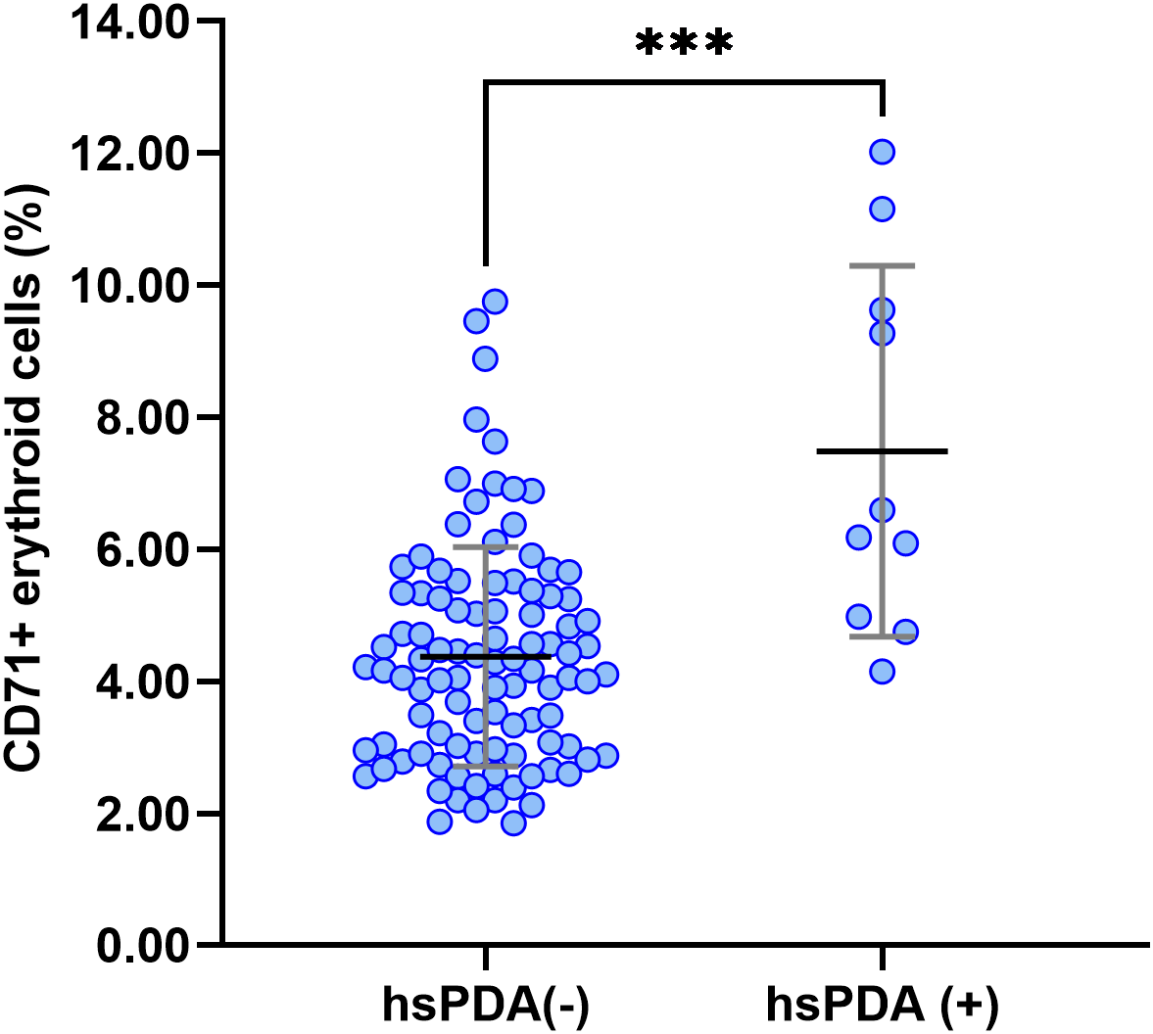
Comparison of CEC levels between preterm infants with and without hsPDA. Infants diagnosed with hsPDA demonstrated significantly elevated CEC levels (mean ± SD; 7.5 ± 2.8%, n = 10) versus those without hsPDA (4.4 ± 1.7%, n = 98), *p* < 0.0001. Each dot represents an individual patient sample (***p < 0.001).

We compared blood test results on the first day of life between preterm infants with and without hsPDA (**Table 2**). Infants with hsPDA showed significantly higher CECs levels (7.5± 2.8 vs. 4.4 ± 1.7, p<0.0001) compared to those without hsPDA (**Figure 3**). Among the 10 infants with hsPDA, we compared CEC levels according to ductal diameter (< 2.5 mm, n = 5, vs. ≥ 2.5 mm, n = 5). The median CEC percentage was 4.98% (interquartile range (IQR) 4.60–7.57) in the smaller PDA group and 9.27% (IQR 6.49–10.00) in the larger PDA group. Although the difference did not reach statistical significance, there was a trend toward higher CEC levels in the larger-PDA group (*p* = 0.151). Conversely, hemoglobin, RBC count, hematocrit, white blood cell count, and platelet count were all significantly lower in the hsPDA group (all *p* < 0.05). In blood gas analysis, PCO_2_ was significantly lower in the hsPDA group, while other parameters, including CRP, did not differ significantly between the groups.

**Table 2:** Comparison of hematologic and clinical parameters by hsPDA status in preterm infants.

Parameter	Total (n=108)	hsPDA (–) (n=98)	hsPDA(+) (n=10)	P-value
Hemoglobin (g/dL)	17.1 ± 2.0	17.2 ± 1.9	15.5 ± 2.2	0.0077
RBC (×10^6^/μL)	4.4 ± 0.6	4.5 ± 0.7	3.9 ± 0.7	0.0007
Hematocrit (%)	49.8 ± 5.9	50.3 ± 5.7	45.5 ± 6.0	0.0107
WBC (/μL)	11,117 ± 6,049	11,585 ± 6,065	6,527 ± 3,478	0.0111
Platelet (×10³/μL)	263.2 ± 72.8	269.1 ± 68.9	205.8 ± 88.3	0.0082
CECs (%)	4.7 ± 2.0	4.4 ± 1.7	7.5 ± 2.8	<0.0001
CRP (mg/dL)	0.1 ± 0.3	0.1 ± 0.3	0.1 ± 0.1	0.7803
pH	7.3 ± 0.1	7.3 ± 0.1	7.3 ± 0.1	0.1748
PO_2_ (mmHg)	58.2 ± 21.7	58.9 ± 22.2	51.3 ± 17.3	0.2923
PCO_2_ (mmHg)	50.2 ± 10.7	51.1 ± 10.6	40.7 ± 9.5	0.0029
SPO_2_ (%)	81.6 ± 14.7	81.8 ± 14.7	79.6 ± 15.1	0.6580
Base deficit (mmol/L)	-4.6 ± 3.5	-4.5 ± 3.6	-5.8 ± 2.4	0.2854

### 3.4 Prediction of hsPDA

**Table 3** presents the results of logistic regression analyses assessing predictors of hsPDA. In univariable analysis, gestational age, birth weight, hemoglobin, RBC count, hematocrit, white blood cell count, platelet count, and CECs were significant predictors of hsPDA (all *p* < 0.05). In the multivariable model, elevated CECs (odds ratio: 2.048, 95% confidence interval: 1.326–3.162, *p* = 0.0012) and lower WBC count (odds ratio: 0.782, 95% CI: 0.620–0.986, *p* = 0.038) remained as independent risk factors for hsPDA. In addition, a *post-hoc* power analysis using G*Power based on the observed effect size for hsPDA (hsPDA n=10 vs. no/restrictive PDA n=98) showed a statistical power of 92.3%, exceeding the conventional 80% threshold. ROC analysis was performed to evaluate the predictive performance of CEC levels for hsPDA. In the overall cohort, CECs yielded an AUC of 0.84 (95% CI 0.72–0.96) with an optimal cut−off value of 6.09%, corresponding to a sensitivity of 0.70 and a specificity of 0.87. In infants with gestational age ≤28 weeks, the AUC was 0.82 (95% CI 0.59–1.00) with a cut−off value of 9.27%, sensitivity 0.67 and specificity 0.91 (**Supplementary Figure S2**).

**Table 3:** Logistic regression analysis identifying risk factors for hsPDA in preterm infants.

Variable	Univariable model OR (95% CI)	Univariable P-value	Multivariable model OR (95% CI)	Multivariable P-value
Gestational age (weeks)	0.640 (0.501, 0.819)	0.0004		
Birth weight (g)	0.997 (0.995, 0.999)	0.0012		
Hemoglobin	0.648 (0.462, 0.909)	0.012		
RBC	0.144 (0.041, 0.507)	0.0025		
Hematocrit	0.870 (0.777, 0.974)	0.0156		
WBC	0.755 (0.610, 0.936)	0.0101	0.782 (0.620, 0.986)	0.038
Platelet	0.988 (0.978, 0.998)	0.014		
CECs (%)	1.867 (1.342, 2.599)	0.0002	2.048 (1.326, 3.162)	0.0012

OR, odds ratio; CI, confidence interval

### 3.5 Correlation of CECs and hematologic parameters

In correlation analysis, hematologic parameters including hemoglobin, RBCs, hematocrit, WBCs, and platelets counts showed a trend of increasing with gestational age, although correlations were weak (R² values 0.1 - 0.3, **Supplementary Figure S1**). Similarly, higher CEC levels were associated with lower values of these hematologic parameters, again with weak correlations (R² values 0.1–0.3, **Supplementary Figure S3**).

## 4 Discussion

This study demonstrates that CECs with immunomodulatory effects decrease with increasing gestational age in preterm infants. Importantly, higher CEC levels were significantly associated with an increased risk of developing hsPDA. These results suggest that CECs may serve as a novel biomarker for early identification of preterm infants at risk for hsPDA.

Previous studies have reported a significant association between perinatal inflammation and PDA in preterm infants (5, 6). However, our study showed no significant differences in CRP levels or in the incidence of maternal chorioamnionitis and PROM between groups. This suggests that traditional inflammatory indices may not fully detect the risk of hsPDA, further emphasizing the value of novel biomarkers such as CECs. Meanwhile, our results about CECs are consistent with recent studies indicating that CECs can exert complex immunomodulatory effects, including promoting pro-inflammatory cytokine release under certain conditions. Miller et al. (17) demonstrated that when neonatal CD71+ erythroid cells come into direct contact with maternal mononuclear immune cells, they induce the release of pro-inflammatory cytokines while decreasing TGF-β secretion. Additionally, in preterm infants born after preterm labor, depletion of CD71+ erythroid cells from cord blood mononuclear cells resulted in decrease in myeloid cell secreting IL-6, IFN-γ, and IL-4 in the presence of microbial products. Furthermore, preterm labor derived CD71+ erythroid cells were shown to enhance early CD8+ T cell activation. These findings suggest that maternal–fetal immune crosstalk can “prime” fetal CECs toward a more pro−inflammatory phenotype, indicating that CECs may not only reflect a state of immunosuppression but also participate in inflammatory processes that could contribute to the persistence of hsPDA. This supports the concept that maternal–fetal immune crosstalk may prime fetal CECs toward a pro−inflammatory phenotype, which in turn could promote the persistence of hsPDA. Further mechanistic studies are necessary to elucidate this pathway, as it remains unclear whether elevated CEC levels are a cause, a consequence, or simply an epiphenomenon of the inflammatory and hypoxic condition associated with hsPDA.

As CD71+ erythroid cells are immature erythrocytes, CECs levels were inversely correlated with hemoglobin and other hematologic parameters, which generally increased with gestational age. This trend explains the significant differences observed between the hsPDA and control groups in hematologic parameters (**Table 2**). In multivariable analysis, CECs remained a robust predictor of hsPDA risk, outperforming hemoglobin, RBC, hematocrit, and platelet levels after adjustment for gestational age (**Table 3**). This suggests that CECs may integrate both hematologic and immunologic alterations linked to hsPDA risk more effectively than individual traditional hematologic indices. Additionally, in a descriptive analysis restricted to infants with hsPDA, higher early CEC levels tended to be observed in those who later demonstrated a larger ductal diameter (≥ 2.5 mm) on echocardiography. Although this trend did not reach statistical significance because of the limited sample size, it raises the possibility that early CEC elevation may be linked to subsequent PDA enlargement, a hypothesis that should be addressed in larger prospective studies. Despite the high post−hoc power (92.3%), the small number of hsPDA events (n=10) and the lower gestational age of infants with hsPDA—given that CEC levels are strongly related to gestation—may still limit the precision and generalizability of the logistic regression estimates, and the results should therefore be interpreted with caution, as a residual risk of type II error cannot be entirely ruled out.

Higher WBC counts were associated with a lower likelihood of hsPDA even after adjusting for gestational age in our cohort. Previous studies have reported inconsistent associations between platelet counts or anemia and hsPDA, and low hemoglobin levels at birth have also been linked to increased mortality in preterm infants (18–21). Because hematologic parameters change with gestational age and are related to morbidity and mortality in preterm infants, larger prospective studies including reticulocyte count are needed to clarify whether these blood cell indices can serve as reliable predictors of hsPDA or related adverse outcomes. Since CECs are immature erythrocytes, their levels tend to increase in cases of anemia, which explains the inverse relationship observed between CECs and hemoglobin. Our study also confirmed that platelet counts negatively correlate with CECs. Although Chen et al. (2014) reported that lower hemoglobin levels increased the risk of hsPDA in VLBW infants, their analysis did not adjust for gestational age (22). In our study, after performing multivariable analysis with adjustment for gestational age, CECs demonstrated a better explanatory power for hsPDA occurrence than hemoglobin. In line with these findings, ROC analysis demonstrated that early CEC measurement has good discriminative ability for predicting hsPDA, with an AUC of 0.84 in the overall cohort and 0.82 in infants ≤28 weeks of gestation. The relatively high specificity, particularly in the very preterm subgroup, suggests that CEC-based stratification could help identify infants at truly high risk for hsPDA and thereby support more targeted use of echocardiography and therapeutic interventions. While this study focused solely on the relationship between CECs and hsPDA, further research is warranted to explore their associations with other morbidities.

Correa-Rocha et al. confirmed that preterm infants exhibit marked leukopenia and lymphopenia (23). Similarly, in our study, WBC counts tended to increase with gestational age, but the correlation was weak. WBC levels are influenced by a wide range of factors; for example, they are naturally higher after vaginal delivery compared to cesarean section (24) and can also increase due to steroid administration or intrauterine stress (25). Conversely, maternal hypertensive disorders and medication use may lead to decreased fetal WBC counts (25, 26). Importantly, even after adjusting for gestational age in the multivariable model, higher WBC counts were significantly associated with a lower likelihood of hsPDA occurrence, which was an unexpected yet interesting finding. The lower hemoglobin, hematocrit, and hematologic parameters observed in infants with hsPDA may contribute to reduced blood viscosity. According to Poiseuille’s law, which relates flow to viscosity, decreased blood viscosity would theoretically increase volumetric flow through the patent ductus arteriosus, promoting hemodynamic significance (27–29). Conversely, the protective association between higher WBC counts and reduced hsPDA risk may reflect the contribution of blood viscosity to ductal hemodynamics, as elevated WBC can increase blood viscosity and reduce ductal shunt volume. These hematologic parameters therefore represent potentially modifiable factors in the hemodynamic pathophysiology of hsPDA. Future studies should explore these subsets in greater depth to better understand which specific immune cell types are associated with hsPDA development.

Our study is the first to present correlation between CEC values and gestational age, and to investigate their association with hsPDA in preterm infants. While echocardiography remains the gold standard for hsPDA diagnosis, its application in very preterm infants is technically challenging and sometimes unreliable due to operator dependency and clinical instability. Early identification of at-risk infants using CECs could enhance neonatal care by informing risk stratification and enabling targeted interventions. BNP and NT proBNP are well validated cardiac biomarkers that correlate with ductal size, shunt magnitude, and the need for hsPDA treatment, and they are already used in clinical practice with established assay platforms and cut off values (30–32). In contrast, CECs reflect endothelial immaturity and activation rather than myocardial wall stress, and in our cohort elevated CEC levels within the first 12 hours after birth preceded the echocardiographic diagnosis of hsPDA, suggesting a role in very early risk stratification before BNP–guided or echocardiographic decision making. Therefore, CECs should not be viewed as a replacement for BNP at this stage but as a complementary biomarker that may help identify high risk infants for targeted echocardiography and subsequent BNP based monitoring, a concept that requires validation in larger prospective studies.

For CECs to be adopted as a biomarker in clinical practice, testing should be feasible within routine neonatal workflows, inexpensive, rapid, and analytically reliable. In this study, CECs were measured using flow cytometry, which offers high analytical specificity but is less practical for universal screening of preterm infants because it requires specialized equipment, trained personnel, and a turnaround time of approximately 24–48 hours. Although flow cytometry reagents add cost, the overall expense is still lower than that of a single echocardiographic examination and becomes more cost-effective when applied at scale. As an instrument−based method, flow cytometry also allows standardization of sample processing, which is expected to yield good reproducibility. For broader clinical implementation, the development of simpler, potentially automated CEC assays would further enhance feasibility. Ongoing work into agents able to modulate CEC expansion further underscores the potential for CEC-directed therapies in the management of hsPDA. By identifying infants with elevated CEC levels, clinicians could potentially tailor pharmacologic or interventional strategies to those at highest risk of hsPDA−related complications. Several studies are currently developing agents to prevent CEC expansion (14), and our research provides a foundational basis suggesting that pharmacological modulation of CECs could serve as a new therapeutic approach for hsPDA in the future.

Several limitations should be acknowledged. This was a single center study conducted in a Korean tertiary NICU with a limited number of hsPDA cases, which constrains generalizability to other racial and ethnic populations and to different clinical practice settings. Because this was an observational study with a small number of hsPDA events, our analyses can only demonstrate associations rather than prove a causal effect of CD71+ erythroid cells on hsPDA outcomes. This study also lacks an external validation cohort, so the predictive performance of CECs may be overestimated. All infants born before 37 weeks’ gestation were analyzed as a single preterm group, which may mask gestational age–specific differences in CEC biology and hsPDA risk. Restricted blood sample volumes precluded cytokine profiling and functional assays, which are needed to directly address the underlying inflammatory mechanisms. Future research using cytokine analysis and measurement of more specific inflammatory markers, including procalcitonin will be essential to substantiate and clarify the immune pathways involved in CEC-mediated ductal patency. CEC levels were measured within the first 12 hours of life, whereas echocardiography for hsPDA was performed later at days 3–7. At birth, despite patent and often large ductal lumens, left-to-right shunting through the ductus arteriosus is minimal because pulmonary vascular resistance remains elevated. Therefore, echocardiographic assessment at birth would not reliably identify hemodynamic significance. By performing echocardiography at days 3–7, we captured the window when ductal hemodynamic effects become clinically relevant and detectable. However, this time difference between CEC measurement and echocardiographic assessment may have influenced our findings and should be considered when interpreting the results. In addition, although CECs and inflammatory markers were sampled at the same time as part of routine NICU blood work, the dynamic nature of postnatal inflammation means that residual timing effects cannot be completely excluded. Finally, we could not fully control perinatal factors that may affect CEC levels, such as the exact timing of cord clamping, the interval between antenatal steroid administration and delivery, and intrauterine hypoxia. Prior studies have shown that delayed cord clamping improves hemoglobin levels and protects against iron-deficiency anemia (33–35). In the present study, none of the infants underwent delayed cord clamping, and the frequencies of antenatal steroid use and hypoxia-related conditions (pregnancy-induced hypertension, placental abruption, meconium staining) were similar between groups. However, these factors may still have affected CEC levels and should be regarded as potential sources of residual confounding. Future multicenter studies with larger cohorts, expanded immunophenotyping, and standardized perinatal practices are needed to validate our findings. In addition, future work should account for the heterogeneity of preterm infants by stratifying them into finer gestational-age and clinical subgroups and by characterizing longitudinal trajectories of CEC levels to refine gestation-specific reference ranges. Applying modern causal-inference approaches, such as target trial emulation, will also be important to determine whether early CEC elevation has a causal impact on hsPDA risk in distinct patient phenotypes.

In summary, CECs decrease with increasing gestational age, and higher CEC levels on the first day of life show a statistically significant association with the subsequent development of hsPDA in preterm infants. These findings suggest that CECs could be used as predictive biomarkers for hsPDA, with potential to improve neonatal outcomes. Further large-scale studies are necessary to confirm these results and to clarify the underlying mechanisms underlying CEC-related risk of hsPDA.

## Data Availability

The original contributions presented in the study are included in the article/**Supplementary Material**. Further inquiries can be directed to the corresponding authors.

## Supplementary material

Supplementary Table 1 Staging system for determining the magnitude of the hemodynamically significant ductus arteriosus, based on echocardiographic criteria. DA Vmax: ductus arteriosus peak velocity, E/A: early passive to late atrial contractile phase of transmitral filling ratio, IVRT: isovolumic relaxation time

Supplementary Figure 1 Scatter plots and regression lines illustrate the correlations between gestational age and various hematologic parameters (hemoglobin, RBC count, hematocrit, WBC count, and platelet count) in preterm infants. All parameters demonstrated a positive but weak correlation with gestational age (R² = 0.1–0.3).

Supplementary Figure 2 Receiver operating characteristic (ROC) curves for early CEC levels in predicting hsPDA. (A) ROC curve for the overall cohort (n = 108), showing an AUC of 0.84 (95% CI, 0.72–0.96) with an optimal cut-off value of 6.09%, corresponding to a sensitivity of 0.70 and a specificity of 0.87. (B) ROC curve for infants with gestational age ≤28 weeks, showing an AUC of 0.82 (95% CI, 0.59–1.00) with an optimal cut-off value of 9.27%, corresponding to a sensitivity of 0.67 and a specificity of 0.91.

Supplementary Figure 3 Scatter plots and regression lines showing the correlations between circulating CD71+ erythroid cell (CEC) percentage and hematologic parameters (hemoglobin, RBC count, hematocrit, WBC count, and platelet count) in preterm infants. Increasing CEC levels were associated with lower values of these parameters, with weak inverse correlations (R² = 0.1–0.3).
