# Supplementary material for: CD71+ erythroid cells as a potential early biomarker for hemodynamic significant patent ductus arteriosus in preterm infants
Source: Front Immunol. 2026 Jan 13;16:1738166. doi: 10.3389/fimmu.2025.1738166 (PMC12834744; doi:10.3389/fimmu.2025.1738166)
Supplement: Supplementary Table 1 — Staging system for determining the magnitude of the hemodynamically significant ductus arteriosus, based on echocardiographic criteria. DA Vmax: ductus arteriosus peak velocity, E/A: early passive to late atrial contractile phase of transmitral filling ratio, IVRT: isovolumic relaxation time [file SupplementaryFile1.docx]

**Supplementary table 1. Staging system for determining the magnitude of the hemodynamically significant ductus arteriosus, based on echocardiographic criteria**

| Staging system for determining the magnitude of the hemodynamically significant ductus arteriosus, based on echocardiographic criteria | |
| --- | --- |
| E1 | No evidence of ductal flow on two-demensional or Doppler interrogation |
| E2 | Small nonsignificant ductus arteriosus   - Transductal diameter < 1.5mm - Restrictive continuous transductal flow (DA V_max_ >2.0 m/s) - No signs of left left heart volume loading (eg. Mitral regurgitant jet > 2.0m/s or LA:Ao ratio > 1.5:1) - No signs of left heart pressure loading (eg, E/A ratio > 1.0 or IVRT > 50) - Normal end-organ (eg, superior mesenteric, middle cerebral) arterial diastolic flow |
| E3 | Moderate HSDA   - Transductal diameter 1.5-3.0mm - Unrestrictive pulsatile transductal flow (DA V_max_ < 2.0 m/s) - Mild-moderate left heart volume loading (eg, LA:Ao ratio 1.5-2:1) - Mile-moderate left heart pressure loading (eg, E/A ratio >1.0 or IVRT 50-60) - Decreased or absent diastolic flow in superior mesenteric artery, middle cerebral artery, or renal artery |
| E4 | Large HSDA   - Transductal diameter > 3.0mm - Unrestrictive pulsatile transductal flow - Severe left heart volume loading (eg, LA:Ao ratio 2:1, mitral regurgitant jet > 2.0 m/s) - Severe left heart pressure loading (eg, E/A ratio > 1.5 or IVRT > 60) - Reversal of end-diastolic flow in superior mesenteric artery, middle cerebral artery or renal artery |

**DA Vmax : ductus arteriosus peak velocity, E/A : early passive to late atrial contractile phase of transmitral filling ratio, IVRT : isovolumic relaxation time**
