# Supplementary figures and images for: CD71+ erythroid cells as a potential early biomarker for hemodynamic significant patent ductus arteriosus in preterm infants
Source: Front Immunol. 2026 Jan 13;16:1738166. doi: 10.3389/fimmu.2025.1738166 (PMC12834744; doi:10.3389/fimmu.2025.1738166)

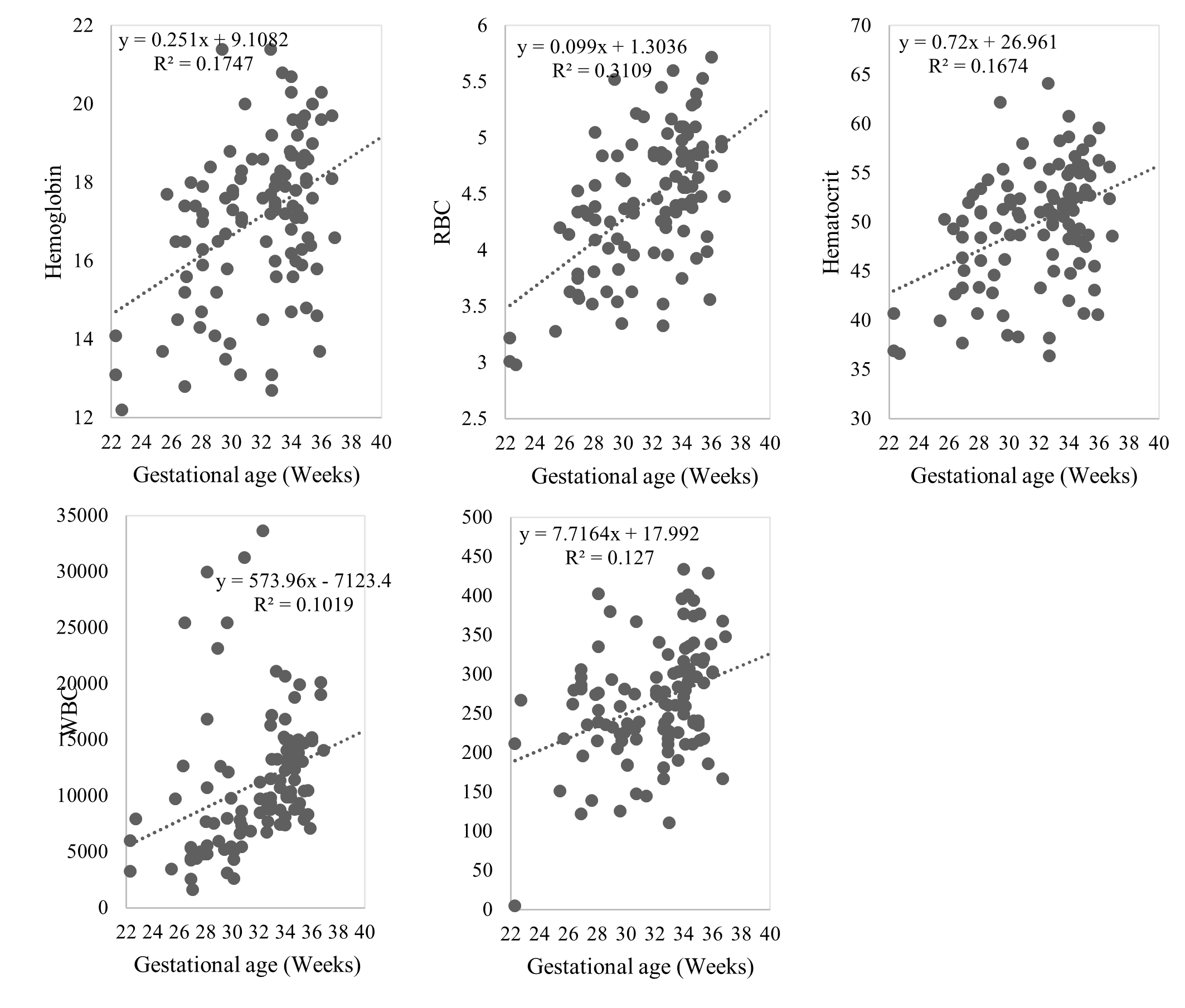

Supplement: Supplementary Figure 1 — Scatter plots and regression lines illustrate the correlations between gestational age and various hematologic parameters (hemoglobin, RBC count, hematocrit, WBC count, and platelet count) in preterm infants. All parameters demonstrated a positive but weak correlation with gestational age (R² = 0.1–0.3). [file Image1.tif]

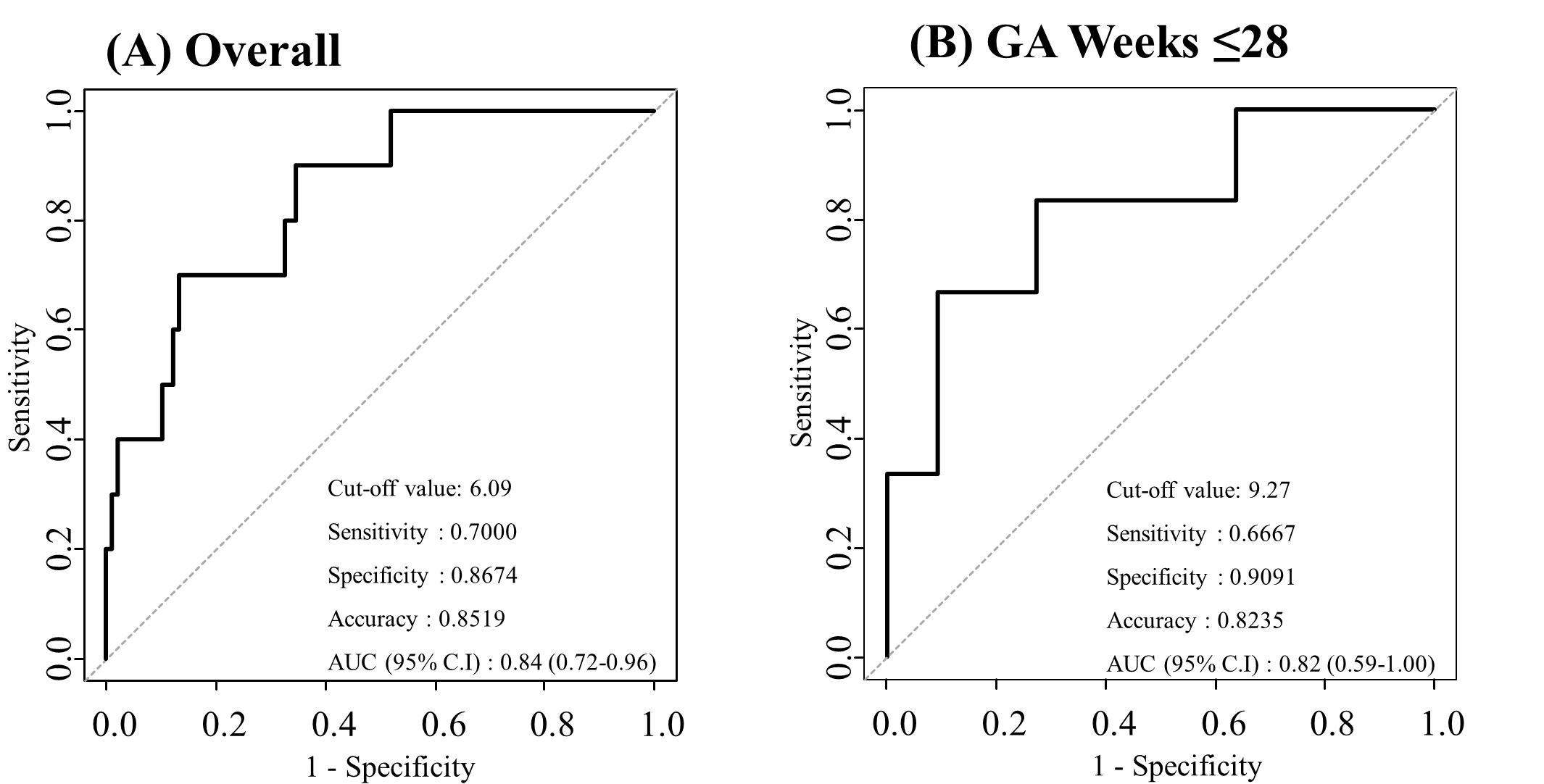

Supplement: Supplementary Figure 2 — Receiver operating characteristic (ROC) curves for early CEC levels in predicting hsPDA. (A) ROC curve for the overall cohort (n = 108), showing an AUC of 0.84 (95% CI, 0.72–0.96) with an optimal cut-off value of 6.09%, corresponding to a sensitivity of 0.70 and a specificity of 0.87. (B) ROC curve for infants with gestational age ≤28 weeks, showing an AUC of 0.82 (95% CI, 0.59–1.00) with an optimal cut-off value of 9.27%, corresponding to a sensitivity of 0.67 and a specificity of 0.91. [file Image2.tif]

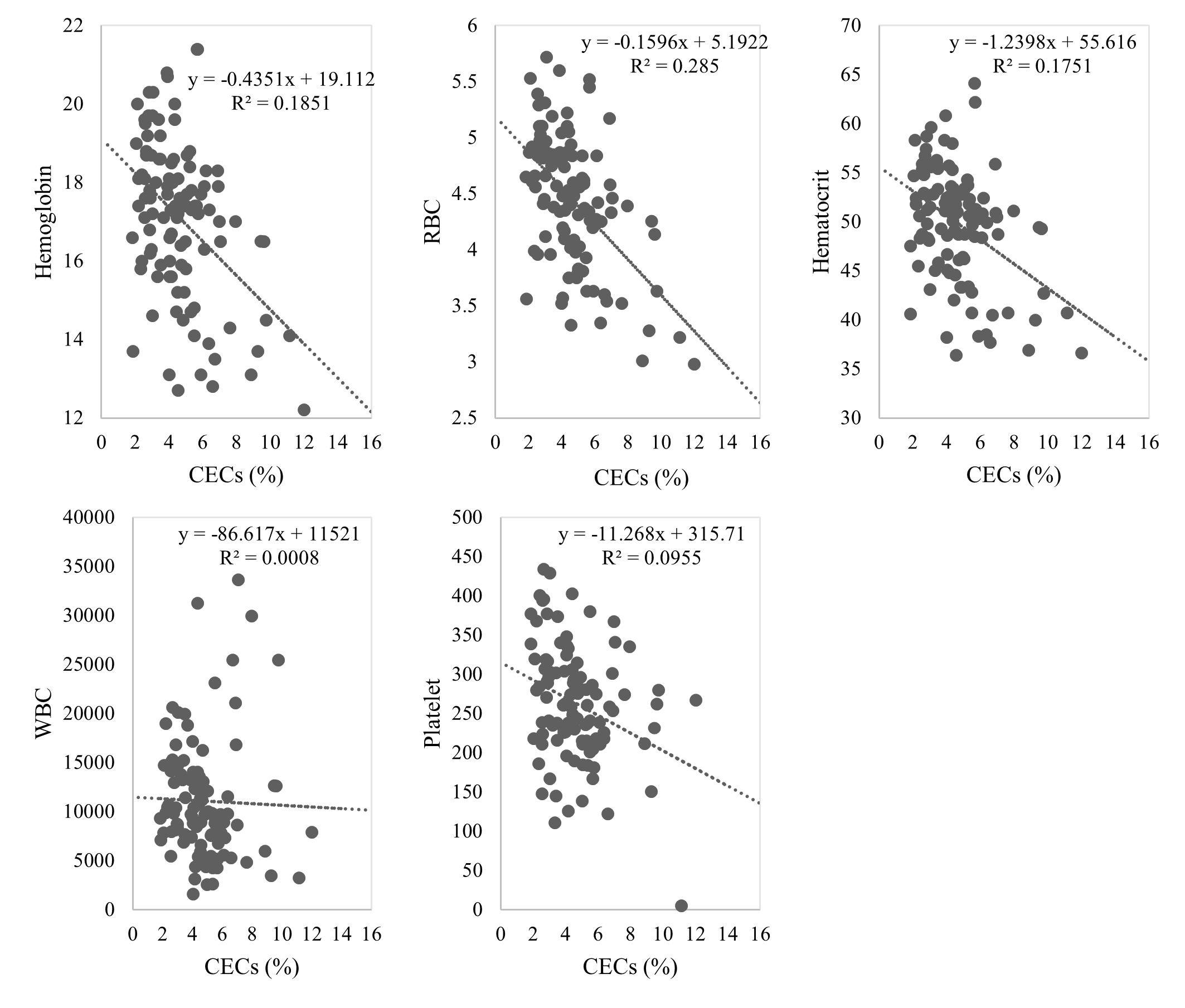

Supplement: Supplementary Figure 3 — Scatter plots and regression lines showing the correlations between circulating CD71+ erythroid cell (CEC) percentage and hematologic parameters (hemoglobin, RBC count, hematocrit, WBC count, and platelet count) in preterm infants. Increasing CEC levels were associated with lower values of these parameters, with weak inverse correlations (R² = 0.1–0.3). [file Image3.tif]
